# Brain-inhabiting bacteria and neurodegenerative diseases: the “brain microbiome” theory

**DOI:** 10.3389/fnagi.2023.1240945

**Published:** 2023-10-19

**Authors:** Tarek Ziad Arabi, Aliyah Abdulmohsen Alabdulqader, Belal Nedal Sabbah, Abderrahman Ouban

**Affiliations:** ^1^College of Medicine, Alfaisal University, Riyadh, Saudi Arabia; ^2^Department of Pathology, College of Medicine, Alfaisal University, Riyadh, Saudi Arabia

**Keywords:** neurodegenerative disease, brain microbiome, Alzheimer’s disease, Parkinson’s disease, bacteria, brain-inhabiting bacteria, fungi, multiple sclerosis

## Abstract

Controversies surrounding the validity of the toxic proteinopathy theory of Alzheimer’s disease have led the scientific community to seek alternative theories in the pathogenesis of neurodegenerative disorders (ND). Recent studies have provided evidence of a microbiome in the central nervous system. Some have hypothesized that brain-inhabiting organisms induce chronic neuroinflammation, leading to the development of a spectrum of NDs. Bacteria such as *Chlamydia pneumoniae*, *Helicobacter pylori*, and *Cutibacterium acnes* have been found to inhabit the brains of ND patients. Furthermore, several fungi, including *Candida* and *Malassezia* species, have been identified in the central nervous system of these patients. However, there remains several limitations to the brain microbiome hypothesis. Varying results across the literature, concerns regarding sample contamination, and the presence of exogenous deoxyribonucleic acids have led to doubts about the hypothesis. These results provide valuable insight into the pathogenesis of NDs. Herein, we provide a review of the evidence for and against the brain microbiome theory and describe the difficulties facing the hypothesis. Additionally, we define possible mechanisms of bacterial invasion of the brain and organism-related neurodegeneration in NDs and the potential therapeutic premises of this theory.

## 1. Introduction

Recently, concerns have risen regarding the validity and, possibly, the falsification of the toxic proteinopathy theory of Alzheimer’s disease (AD) (Espay et al., 2023). Therefore, many among the scientific community have begun the search for an alternative hypothesis. Recent studies have postulated that specific microorganisms in the brain microbiome induce chronic neuroinflammation leading to AD.

The brain microbiome refers to the complex community of microorganisms, including bacteria, viruses, fungi, and other microbes, that reside within the human brain (Link, 2021). Traditionally, the brain was thought to be a sterile environment devoid of any microbial presence. However, recent scientific investigations have challenged this paradigm and shed light on the potential existence of a diverse and dynamic microbiota within the brain (Link, 2021).

Understanding the involvement of a brain microbiome has profound implications for human health. The brain-gut axis, a bidirectional communication pathway between the brain and the gut microbiome, highlights the potential interactions and influence of bacteria on brain function and behavior (Martin et al., 2018). Microbial metabolites, neurotransmitters, and immune molecules produced by the gut microbiota can influence brain function and behavior through various signaling pathways (Martin et al., 2018). Conversely, the brain can also exert control over the gut microbiota through neural, endocrine, and immune mechanisms (Galland, 2014). Alterations in the brain microbiome could have implications for neurological disorders.

Molecular techniques, such as high-throughput deoxyribonucleic acid (DNA) sequencing and metagenomic analysis, have provided valuable insights into the microbial communities present in various body sites (Link, 2021). These methods have found bacterial DNA in samples of brain tissue, proving that there are bacteria inside the brain. In HIV patients, α-proteobacteria were identified throughout all parts of the brain. This was further verified via the usage of both 16S ribosomal ribonucleic acid (RNA) gene amplification and *in-situ* labeling for bacterial peptidoglycans (Branton et al., 2013; Galland, 2014). The statement that the brain is not sterile is strengthened by the several verification techniques performed in these experiments; however, these findings were later on challenged by papers that reflected on the experiments previously performed (Salter et al., 2014; Mangul et al., 2018; Link, 2021). Animal studies have demonstrated the colonization of the brain by bacteria (Roberts et al., 2018). For example, studies using germ-free mice, which lack any resident microbiota, have shown that the introduction of specific bacteria can lead to colonization of the brain (Banerjee et al., 2011). These investigations have demonstrated the colonization of the brain by specific microorganisms, indicating that these microbes can establish and persist within the brain.

The implications of the brain microbiome extend beyond basic microbial colonization. Dysbiosis, an imbalance or alteration in the composition of the brain microbiota, has been associated with several neurological and neuropsychiatric disorders, including AD, multiple sclerosis (MS), Parkinson’s disease (PD), depression, and anxiety (Westfall et al., 2020; Góralczyk-Bińkowska et al., 2022; Nandwana et al., 2022). Understanding the complex interactions between the brain and its resident microorganisms may provide novel insights into the etiology, progression, and potential therapeutic interventions for these conditions.

It is important to note that the concept of the brain microbiome is still in its early stages, and many aspects remain to be elucidated. The field faces challenges such as sample contamination, technical limitations, and methodological differences, which need to be addressed to ensure the reliability and validity of research findings. Although a large amount of data have described an association between inflammation of the gut-brain-microbiome axis and neurodegenerative diseases, this review focuses on direct inhabitation of bacteria and fungi in the central nervous system. We summarize the literature supporting and disproving the presence of a brain microbiome in diseased brains. Then, we highlight possible mechanisms of microbial invasion and disease progression in such patients. Lastly, we describe the limitations of the brain microbiome theory and future research directions. Nevertheless, the emerging evidence supporting the presence of a brain microbiome represents a paradigm shift in our understanding of the brain and its intricate relationship with microorganisms.

## 2. Neurodegenerative diseases and the brain microbiome

Several studies have linked specific bacteria and fungi to the development of neurodegenerative diseases. In this section, we highlight the available studies which support the theory of the brain microbiome and its role in neurodegenerative diseases and describe the evidence against it (Table 1).

**Table 1:** A summary of the evidence supporting and disproving the brain microbiome theory in neurodegenerative disease.

Disease	Organism	Evidence in support	Evidence against
AD	*Chlamydia pneumoniae*	(Balin et al., 1998; Gérard et al., 2006)	(Ring and Lyons, 2000; Taylor et al., 2002; Wozniak et al., 2003)
	*Borrelia burgdorferi*	(MacDonald, 1986; MacDonald and Miranda, 1987; MacDonald, 1988; Senejani et al., 2022)	(Gutacker et al., 1998; McLaughlin et al., 1999)
	*Helicobacter pylori* (no evidence in brain tissues)	(Kountouras et al., 2009; Mawanda and Wallace, 2013; Xie et al., 2023)	(Shiota et al., 2011)
	*Cutibacterium acnes*	(Emery et al., 2017)	None
	*Alternaria*	(Alonso et al., 2018)	
	*Botrytis*	(Alonso et al., 2018)	
	*Candida*	(Pisa et al., 2015; Alonso et al., 2018)	
	*Malassezia*	(Pisa et al., 2015; Alonso et al., 2018)	
	*Cladosporium*	(Pisa et al., 2015)	
	*Neosartorya hiratsukae*	(Pisa et al., 2015)	
	*Phoma*	(Pisa et al., 2015)	
	*Sacharomyces cerevisae*	(Pisa et al., 2015)	
	*Sclerotinia borealis*	(Pisa et al., 2015)	
PD	*Streptococcus*	(Pisa et al., 2020)	(Bedarf et al., 2021)
	*Psueodomonas*		
	*Cutibacterium acnes*		
	*Borrelia burgdorferi*	(Senejani et al., 2022)	
	*Botrytis*	(Pisa et al., 2015, 2020)	
	*Candida*	(Pisa et al., 2020)	
	*Fusarium*	(Pisa et al., 2020)	
	*Malassezia*	(Pisa et al., 2020)	

### 2.1. Alzheimer’s disease and the brain microbiome

Several studies have found exponentially higher levels of bacteria in AD patients compared to control patients (Emery et al., 2017). Among those bacteria, *Chlamydia pneumoniae* has been frequently found in the brains of AD patients (Balin et al., 1998; Gérard et al., 2006). For example, Gerard et al. detected high levels of *C. pneumoniae* DNA in the astrocytes, microglia, and neurons of AD patients, adjacent to amyloid plaques and neurofibrillary tangles (Gérard et al., 2006). On the other hand, others have failed to demonstrate an association between AD and *C. pneumoniae* (Ring and Lyons, 2000; Taylor et al., 2002; Wozniak et al., 2003).

*Borrelia burgdorferi* is another bacterium which authors have identified as a possible microbial cause of AD. Macdonald et al. identified the bacterium in AD postpartum biopsies on several occasions (MacDonald, 1986; MacDonald and Miranda, 1987; MacDonald, 1988). *B. burgdorferi* also co-localizes with amyloid and tau aggregates in patients with AD and PD (Senejani et al., 2022). In an analysis by Miklossy, *B. burgdorferi* was found to be 13-fold more prevalent in AD than control subjects (Miklossy, 2011). Similar to the case of *C. pneumoniae*, some studies have found no association between the microorganism and the pathogenies of AD (Gutacker et al., 1998; McLaughlin et al., 1999). For example, Gutacker et al. found no evidence of the spirochete among AD patients in their study (Gutacker et al., 1998). It is believed that *B. burgdorferi* triggers astrocytes and neurons to release pro-inflammatory cytokines including interleukins (IL)-6, -8, -10, interferon-γ, and tumor necrosis factor (TNF)-ɑ and modulates oxidative stress (Wawrzeniak et al., 2020; Senejani et al., 2022).

Recent evidence has also highlighted the role *Helicobacter pylori*, a Gram-negative bacteria known for chronic gastritis, peptic ulcer disease, and gastric cancer, in the pathogenesis of AD (Mawanda and Wallace, 2013). Kountouras et al. found significantly greater levels of anti-*H. pylori*-specific IgG levels in the cerebrospinal fluid of AD patients than controls (Kountouras et al., 2009). Additionally, the antibody levels positively correlated with disease severity. To the best of our knowledge, there are no studies analyzing the presence of *H. pylori* in postpartum brain tissues of AD patients. Contrarily, Shiota et al. found no association between *H. pylori* status and AD in a study of 917 Japanese patients (Shiota et al., 2011). The mechanisms connecting *H. pylori* and AD remain unclear. Recently, Xie et al. revealed that *H. pylori*-derived outer membrane vesicles (OMVs) in mice gut are capable of crossing biologic barriers via transcellular pathways, eventually traversing the blood–brain barrier and reaching the brain (Xie et al., 2023). In the central nervous system, OMVs enter astrocytes, activate glial cells, and result in neuronal dysfunction. Additionally, the authors demonstrated that complement component 3 (C3) and its receptor signaling plays a key role in mediating the OMV-induced interactions between astrocytes, glial cells, and neurons (Xie et al., 2023). Pharmacological inhibition of the complement pathway shunted OMV-induced glial and neuronal dysfunction and prevented AD pathology and cognitive decline; however, no improvement in astrocyte function was noted (Xie et al., 2023). Whether the positive effects seen with C3 inhibition translate clinically remains unstudied.

In regards to fungi, Alonso et al. demonstrated that fungi of the genera *Alternaria*, *Botrytis*, *Candida*, and *Malassezia* are more common in the frontal cortices of AD patients compared to controls (Alonso et al., 2018). Additionally, Pisa et al. identified fungal material, such as *Candida*, *Cladosporium*, *Malassezia*, *Neosartorya hiratsukae*, *Phoma*, *Sacharomyces cerevisae*, and *Sclerotinia borealis*, in the brain samples of all AD patients in their cohort (Pisa et al., 2015). Notably, no single species was identified in all of the studied regions in the study.

Emery et al. found a significantly increased amount of bacteria associated with the skin, nasopharyngeal, and oral areas (specifically *Cutibacterium acnes*) in postpartum AD patients compared to non-diseased patients (Emery et al., 2017). Although there seems to be a strong connection between brain-inhabiting bacteria and AD, there remains a high level of heterogeneity between studies (Table 1). Further studies are needed to confirm the role of bacteria and fungi in AD and identify key players in the brain microbiome.

### 2.2. Parkinson’s disease and the brain microbiome

Data surrounding the brain microbiome and its association with PD are much more limited than AD (Table 1). Pisa et al. first demonstrated the presence of bacteria in the central nervous system of PD patients (Pisa et al., 2020). The authors found that the bacterial genera *Streptococcus* was frequently present in the main cortex of patients, while *Pseudomonas* commonly inhabited the medulla. Interestingly, *C. acnes* was frequently identified in several patients across different CNS regions, possibly revealing the role of *C. acnes* in neurodegenerative diseases as a whole (Pisa et al., 2020). Additionally, fungi from the genera *Botrytis*, *Candida*, *Fusarium*, and *Malassezia* were seen in the PD specimens (Pisa et al., 2020). However, Bedarf et al. raised concerns regarding the validity of the microbial theory in the development of PD and the presence of a brain microbiome in normal humans, citing off-target amplifications as the reason for large false-positive results in previous studies (Bedarf et al., 2021). Hence, the true role of microorganisms in PD remains unclear.

### 2.3. Amyotrophic lateral sclerosis and the brain microbiome

Amyotrophic lateral sclerosis (ALS) is the most common motor neuron disease worldwide and is associated with rapid mortality within 5 years of diagnosis (Beauverd et al., 2012; Alonso et al., 2019b). Although studies assessing microorganisms in ALS patients are limited, studies demonstrating the presence of a brain microbiome in such patients are emerging. For example, Alonso et al. detected a significantly greater amount of fungal antigens in the cerebrospinal fluid of ALS patients than controls, with an odds ratio of 4.8 (Alonso et al., 2015). Several fungal antigens were visualized in the brains of ALS patients using immunohistochemistry. Using polymerase chain reactions (PCR), the authors extracted *C. albicans* DNA was extracted from all ALS patients analyzed. DNA of *Trichoderma viride* and *Cryptococcus magnus* were also extracted (Alonso et al., 2015). Another study by Alonso et al. supported these findings and further identified fungal DNA from the genera *Malassezia*, *Fusarium*, and *Botrytis* (Alonso et al., 2017).

Then, Alonso et al. attempted to identify bacterial infections in the brain samples of ALS patients (Alonso et al., 2019b). Using nested PCR, several bacteria were detected in ALS central nervous tissue, mainly *C. acnes*, followed by *Corynebacterium*, *Fusobacterium nucleatum*, *Lawsonella clevelandesis*, and *Streptococcus thermophilus*. The bacterial orders *Actinomycetales*, *Burkholderialems*, and *Rhizobiales* were detected in all ALS patients, and *Xanthomonadales* was only detected in two using next-generation sequencing. It has been hypothesized that bacterial and fungal infiltration in ALS promotes neuroinflammation, which is consistent with other studies highlighting the role of neuroinflammatory processes in diseased brains (Henkel et al., 2004; Alonso et al., 2019b). Studies from different groups, however, are needed to confirm these findings.

### 2.4. Multiple sclerosis and the brain microbiome

Multiple sclerosis (MS) is an autoimmune disease commonly affecting the adolescent and young adult populations (Dobson and Giovannoni, 2019). Kriesel et al. aimed to uncover the presence of bacteria in the brains of living MS patients compared to epilepsy patients (control) (Kriesel et al., 2019). Microbial sequence reading was significantly higher in MS patients compared to their control. Additionally, 29 bacterial genera candidates were identified from 11 phyla. Interestingly, one patient was biopsied twice in the study at two different timepoints during the patient’s clinical course. The first sample had a limited number of bacterial and was nearly identical to control specimens. On the other hand, the second biopsy revealed several bacterial candidates. Notably, this study utilized specimens from living patients, limiting the role of postmortem contamination that is commonly seen in other studies. Branton et al. also identified a greater presence of *Proteobacteria* in the white matter of progressive MS patients, associated with increased inflammatory markers, compared to those with relapsing-remitting subtype (Branton et al., 2016). Additionally, studies have shown that non-*albicans Candida* species (*C. glabrata*, *C. krusei*, and *C. parapsilosis*) are able to reach the central nervous system in animal models of MS (Fraga-Silva et al., 2022). Specifically, *C. glabrata* and *C. krusei* exacerbated MS severity, while *C. parapsilosis* did not. Collectively, these findings allude to a strong association between the presence of a brain microbiome with increased progression of MS.

### 2.5. Huntington’s disease and the brain microbiome

Huntington’s disease is a genetic neurodegenerative disease caused by CAG trinucleotide repeat expansion in the Huntington gene (Stoker et al., 2022). Alonso et al. found fungi of the genera *Candida*, *Davidiella*, *Malassezia*, *Rhodotorula*, and *Ramularia* using next-generation sequencing (Alonso et al., 2019a). Several bacteria were also identified, including *Pseudomonas*, *Cutibacterium*, *Escherichia coli*, *Acinetobacter*, and *Burkholderia* in the striata and frontal cortices of these patients (Alonso et al., 2019a). To date, no other studies have attempted to study the brain microbiome in Huntington’s disease.

## 3. How do bacteria reach the brain?

Microbes can enter the brain through hematogeneous spread, which is one of the most common pathways observed in neurological infections. In this process, the organisms infiltrate the bloodstream, and from there, they traverse the blood–brain–barrier (BBB) to reach the brain (Khan et al., 2002; Dando et al., 2014; Espinal et al., 2022). Then, they directly invade the brain by exploiting an impaired BBB via extracellular DNA and lipopolysaccharides (LPS). If the integrity of the BBB is compromised, either due to injury, inflammation, or other underlying conditions, bacteria and fungi can penetrate the barrier and gain access to the brain (Tetz, 2022). Additionally, bacterial extracellular DNA and LPS, which are released by bacteria during infection, can directly disrupt the BBB and facilitate bacterial entry into the brain (Tetz, 2022). This theory underscores the significance of a robust BBB in preventing microbial invasion and highlights the critical role of its compromise in facilitating brain infections.

Another mode of bacterial entry involves the invasion of brain microvascular endothelial cells (BMECs). These cells line the blood vessels within the brain and form a crucial component of the BBB. Research has demonstrated that certain bacteria can induce a process called macropinocytosis in BMECs. Macropinocytosis involves the non-specific uptake of extracellular fluid and solutes, and when activated by bacteria, it enables their internalization into the endothelial cells (Loh et al., 2017; Espinal et al., 2022). This mechanism could provide a direct route for bacteria to breach the BBB and enter the brain parenchyma.

Remarkably, in certain cases, bacteria can also invade the fetal brain following maternal hypoxia. Maternal hypoxia refers to a condition characterized by a decreased oxygen supply to the mother, which can impact the developing fetus. Studies have shown that certain bacteria can cross the placenta and infiltrate the fetal brain during maternal hypoxia, leading to adverse neurological outcomes (Zarate et al., 2017). This pathway highlights the vulnerability of the developing brain and emphasizes the potential consequences of bacterial infections during pregnancy.

Another proposed theory is the invasion of the central nervous system via the olfactory bulb (Pisa et al., 2020). The olfactory bulb is the most external part of the central nervous system and directly connects the external environment with the brain. One of the earliest signs of PD is anosmia as a result of degeneration of the olfactory bulb due to ɑ-synuclein-related pathology and neurotransmitter alterations (Doty, 2012). This theory provides an explanation for the presence of *C. acnes*, a major constituent of the skin flora, in both AD and PD brains (Emery et al., 2017; Pisa et al., 2020). However, further studies are needed to confirm this theory, and evidence directly supporting it are not yet available.

Furthermore, bacterial peptidoglycan, a component of bacterial cell walls, has been found to mediate gut-brain communication through the Nod2 receptor. The Nod2 receptor is present in both the gut and the brain and plays a role in immune regulation. Activation of the Nod2 receptor by bacterial peptidoglycan can initiate signaling pathways that affect brain function and communication (Gabanyi et al., 2022). This highlights the intricate relationship between the gut microbiota and the brain and suggests a potential pathway through which bacteria can indirectly influence brain health and function.

Lastly, the modulation of the microbiota-gut-associated lymphoid tissue (GALT)-brain axis by prebiotics has been shown to indirectly impact the brain. Prebiotics are dietary fibers that promote the growth and activity of beneficial gut bacteria. By positively influencing the gut microbiota composition, prebiotics can have downstream effects on the GALT and subsequently affect brain function and behavior (Elena et al., 2019). This theory highlights the potential of modulating the gut microbiota as a strategy to indirectly mitigate the risk of bacterial invasion and associated neurological complications.

**Figure 1:**
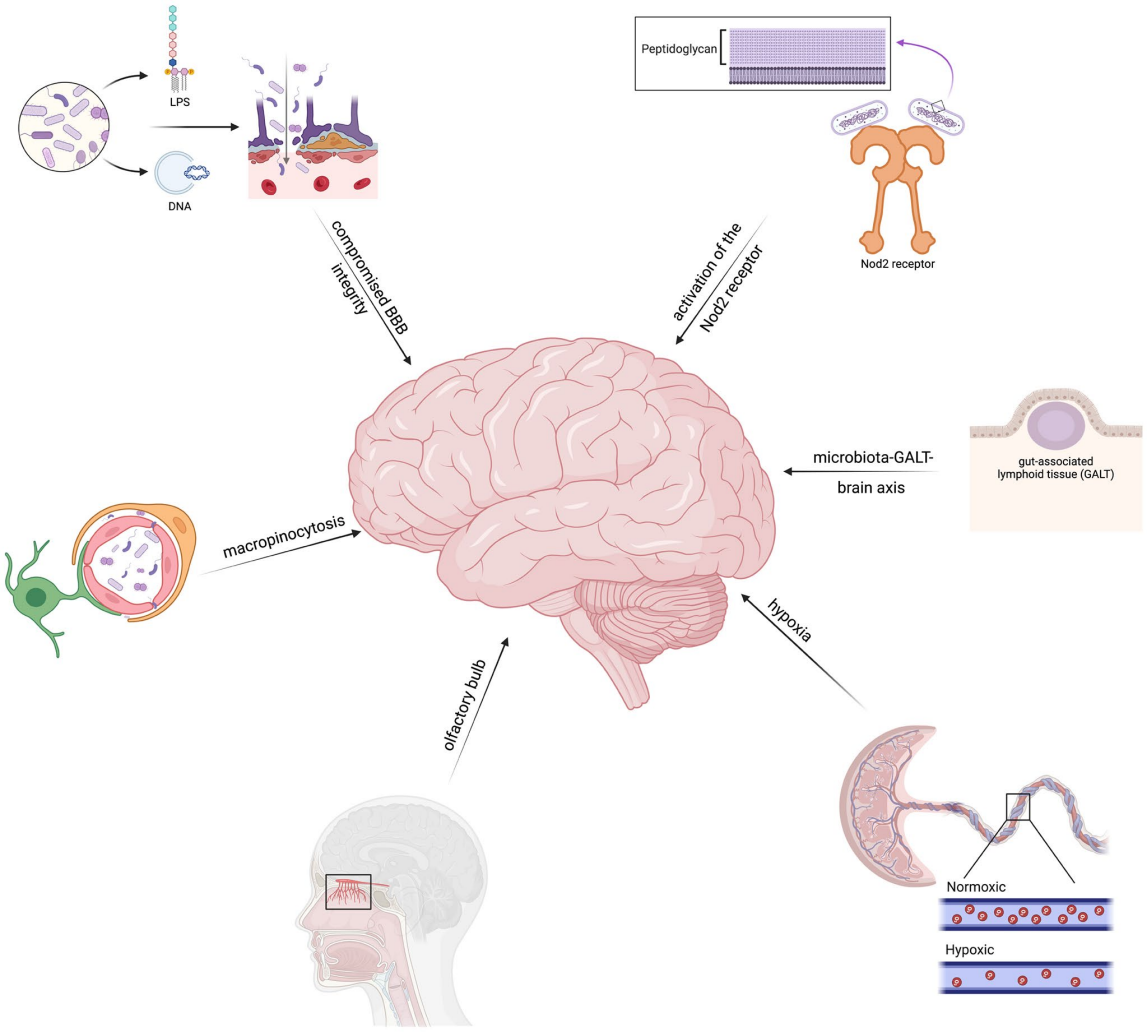
Theories regarding the mechanisms of microbial entry include hematogeneous spread, brain microvascular endothelial cell (BMEC) invasion, abnormal blood–brain–barrier function, placental hypoxia, and gut-brain pathways.

In summary, possible theories for microbial invasion include hematogeneous spread, invasion of BMECs, impairment of the BBB, maternal hypoxia, and gut-brain communication pathways (Figure 1). Understanding these mechanisms is crucial in comprehending how they contribute to the development of neurodegenerative diseases. This knowledge provides valuable insights into the tactics used by bacteria to breach the brain’s defenses and initiate neurological damage associated with neurodegenerative diseases.

## 4. Mechanisms of microorganism-related neurodegeneration

The key mechanism underlying neurodegeneration in the context of the brain microbiome is neuroinflammation. Proinflammatory cytokines, such as TNF-α and ILs-1β and -6, are significantly elevated in patients with neurodegenerative conditions (Fillit et al., 1991; Liu et al., 2022). Specifically, studies have found elevated IL-6 levels in PD patients with infectious burden consisting of *B. burgdorferi*, *C. pneumoniae*, and *H. pylori* (Bu et al., 2015). Bacterial components, primarily LPS, can induce proinflammatory phenotypic changes of inflammatory cells in the central nervous system. LPS promotes *nuclear factor kappa-light chain enhancer of activated B-cell (NFκB)* gene expression, which subsequently leads to the release of a wide spectrum of cytokines (Hernandez Baltazar et al., 2020). LPS also induces a metabolic switch to glycolysis via mitochondrial splitting in microglial cells (Nair et al., 2019). Targeting LPS-related pathways with dasatinib, a tyrosine kinase inhibitor, has shown promising outcomes in mice. Ryu et al. found that dasatinib significantly regulated LPS-induced changes in microglia and astrocytes, blunted the release of proinflammatory cytokines, and inhibited neutrophil rolling in the central nervous of treated mice (Ryu et al., 2019). Phagocytosis also plays a major role in neuroinflammatory processes. Specifically, spirochetes, such as *B. burgdorferi*, are recognized by toll-like receptors on microglial cells, subsequently triggering a proinflammatory response (Dehhaghi et al., 2018). Additionally, spirochetes can alter coagulation pathways by activating plasminogen and factor XII (Dehhaghi et al., 2018).

**Figure 2:**
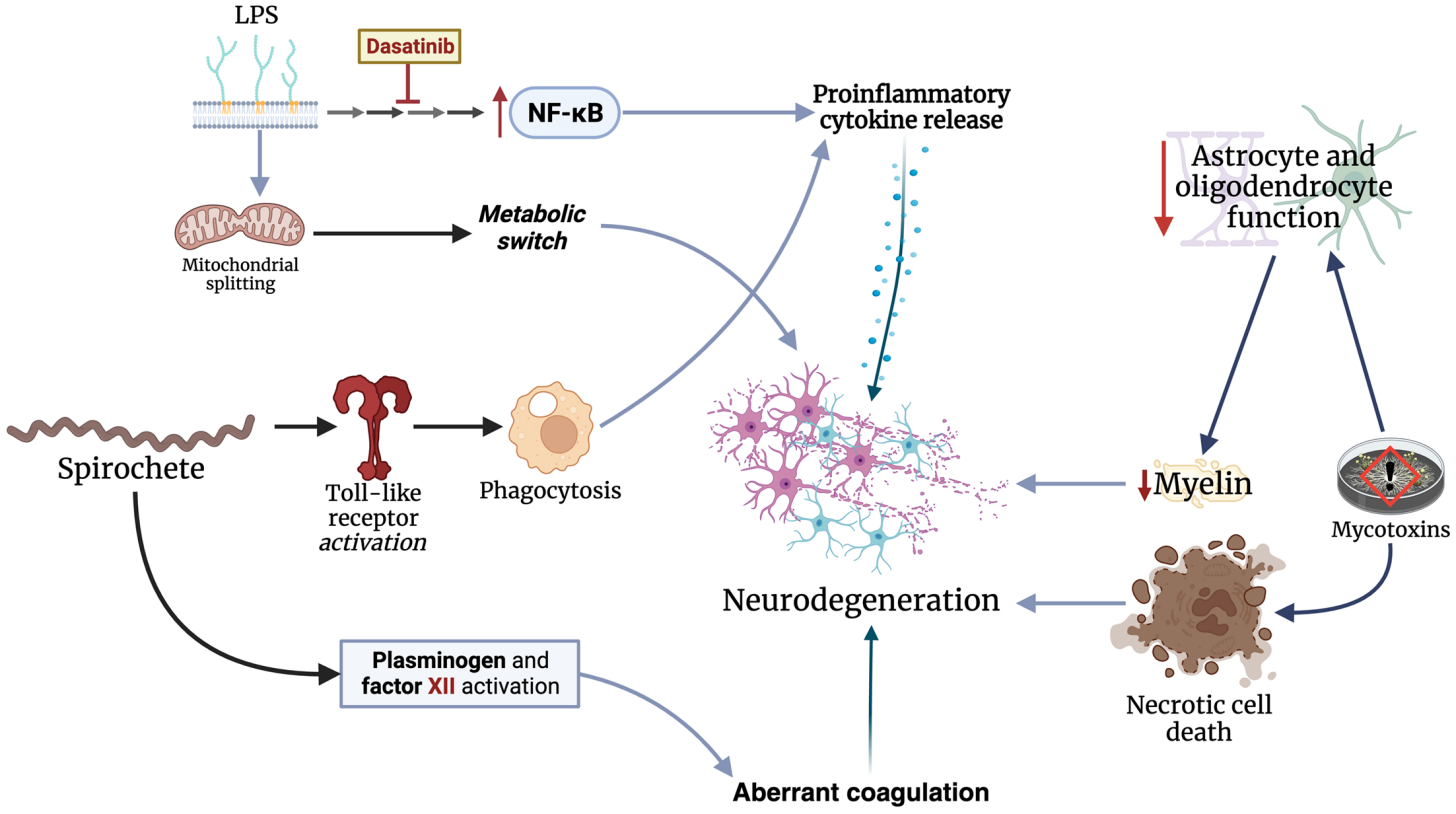
Microbes invading the central nervous mainly through neuroinflammation and direct toxicity by releasing neurotoxic materials.

Besides the proinflammatory mechanisms triggered by brain microorganisms, these microorganisms also have direct neurotoxic effects which promote neurodegeneration. For example, mycotoxins from *Candida* and *Aspergillus* fungi can attach to non-neuronal tissues and continuously release mycotoxins (Purzycki and Shain, 2010). These mycotoxins blunt the function of astrocytes and oligodendrocytes, which maintain the BBB and provide nutrients for myelin production, predisposing patients to MS (Purzycki and Shain, 2010). Additionally, fumonisin B1, a toxin produced by *Fusarium verticillioides*, triggers necrotic cell death and inhibits mitochondrial activity in the central nervous system cells of mice (Osuchowski and Sharma, 2005). However, these effects do not appear to impact primary cortical neurons. Similarly, penitrem A, released by *Penicillium crustosum*, induces tremors in inflicted rats as a result of ischemic cell changes in their cerebella (Cavanagh et al., 1998). In conclusion, microorganisms can promote neurodegeneration through several mechanisms, primarily through neuroinflammation and direct neurotoxic effects (Figure 2).

## 5. The brain microbiome as a therapeutic target

The question of whether these organisms can be targeted with antibiotics arises in light of the increasing body of research indicating the existence of a brain microbiome. Due to the impermeability of the BBB, only a select number of antimicrobials, such as metronidazole, some fluoroquinolones, and fluconazole, are capable of reaching the central nervous system (Nau et al., 2010). However, there are no reports directly addressing the effectiveness of antimicrobials in treating and preventing neurodegenerative diseases in the context of the brain microbiome. Nevertheless, several trials have studied the impact of antimicrobials on the outcome of neurodegenerative diseases.

The use of tetracyclines has been studied in AD and Huntington’s disease. For example, minocycline prevents disease progression in Huntington’s disease and ALS mice models (Chen et al., 2000; Zhang et al., 2003). In support of these findings, a meta-analysis on the impact of minocycline in rodent models of neurodegenerative disease found that the drug has neuroprotective effects in such models (Li et al., 2013). Although the antibiotic has shown a safe and tolerable profile in Huntington’s disease patients (Thomas et al., 2004), a clinical trial conducted in 2010 demonstrated futility of the treatment (Huntington Study Group DOMINO Investigators, 2010). Ceftriaxone, a third-generation cephalosporin, has also been studied in ALS patients. Although stage 1 and 2 clinical trials showed a promising safety profile (Berry et al., 2013), a randomized, controlled phase 3 trial failed to demonstrate any impact on disease (Cudkowicz et al., 2014). Recently, a randomized, controlled trial by Mandrioli et al. demonstrated that rapamycin has no impact on disease progression, survival, or quality of life in ALS patients (Mandrioli et al., 2023). Although antimicrobials have proven effective in preclinical models, it appears that these results fail to translate to the clinical field. Nevertheless, more trials are needed to determine if there are any unstudied antimicrobials capable of altering the brain microbiome and whether such effects can blunt disease progression.

## 6. Future research directions

Recent research has delved into the intricate relationship between the human microbiome and human health. While the gut microbiome has received significant attention, new evidence suggests its impact extends beyond digestion. The exploration of the brain microbiome has revealed a previously overlooked aspect of the microbiota-brain axis, leading to inquiries about its potential implications for neurodegenerative diseases. Unraveling the role of the brain microbiome in diseases like AD and PD could provide innovative treatment targets and diagnostic methods, shaping the future of clinical practice. In this section, we explore the potential future research directions in studying the brain microbiome and its possible contributions to neurodegenerative diseases.

One promising avenue for investigation is to further characterize the composition and dynamics of the microbiota present in the brain. The spectrum of brain microbes differs between individuals, as well as between brain regions, examined from single individuals (Hu et al., 2023). Significant differences in beta diversity have been noted between samples obtained from the hippocampus and cerebellum, underscoring the influence of brain region on the occurrence of microbial DNA (Westfall et al., 2020). Advanced techniques such as metagenomics, transcriptomics, and proteomics can help unravel the intricate microbial ecosystems within the brain (Baldrian and López-Mondéjar, 2014). Deciphering the specific microbial signatures associated with different neurodegenerative diseases holds the potential to provide invaluable insights into the underlying disease mechanisms. By identifying distinct microbial profiles or dysbiotic patterns associated with specific neurodegenerative conditions, researchers can enhance early detection and diagnosis, as well as develop targeted interventions tailored to individual patients.

In addition to understanding the microbial compositions, researchers should also investigate the functional consequences of the brain microbiome in neurodegenerative diseases. This involves exploring how specific microbial species or their metabolites impact the pathology, progression, and symptomatology of these disorders. Microbes produce a wide range of metabolites, such as short-chain fatty acids (SCFAs), neurotransmitters, and secondary metabolites, which can have direct or indirect effects on brain health (Silva et al., 2020). SCFAs such as acetate, propionate, and butyrate not only have local effects in the colon and peripheral tissues, but are also believed to play a crucial role in the communication between the microbiota, gut, and brain. The presence of monocarboxylate transporters in endothelial cells suggests that SCFAs may cross the blood–brain barrier, as previous studies have demonstrated the uptake of SCFAs in the brain of rats through carotid artery injection. However, the existing research on the physiological concentrations of SCFAs in the brain and other microbial metabolites is limited (Silva et al., 2020).

It is imperative to deepen our understanding of how these metabolites influence crucial processes like neuroinflammation, protein aggregation, oxidative stress, and other factors associated with neurodegeneration. Expanding knowledge in these areas will contribute significantly to unraveling the complexities of neurodegenerative diseases and developing effective therapeutic interventions.

## 7. Limitations to the brain microbiome theory

It is important to note that the presence of a brain microbiome and, accordingly, the role of microorganisms in neurodegenerative diseases remain highly controversial. The varying results across AD and PD patient populations have risen doubts surrounding the brain microbiome theory. As previously stated, Bedarf et al. found no evidence for microorganisms in the brains of both PD and control patients (Bedarf et al., 2021). Similar findings can be seen in AD, where several studies have found no correlation between the presence of CNS-inhabiting bacteria and AD (Ring and Lyons, 2000; Taylor et al., 2002; Wozniak et al., 2003). Hence, while the brain microbiome theory may provide a plausible alternative to the toxic proteinopathy hypothesis, caution must be taken when interpreting the results of previous studies until greater data emerge.

One of the main challenges is the potential for contamination from exogenous DNA, which can lead to false-positive microbial signals. Contamination can originate from various sources, including reagents used in sample preparation and sequencing (Link, 2021). Another study highlights the prevalence of substantial amounts of human genomic DNA as an additional factor contributing to contamination, potentially leading to inaccurate outcomes (Emery et al., 2017).

Moreover, the bacterial biomass in brain samples is notably low, presenting a challenge when it comes to detecting and characterizing authentic microbial signals amid the prevalent background noise. The limited microbial biomass complicates the differentiation between genuine microbial presence and potential artifacts introduced during sample processing or sequencing (Bedarf et al., 2021). Another limitation is the potential for false-positive amplification of host DNA, leading to misclassification of host sequences as microbial. This can occur due to off-target amplification or the presence of host DNA in the samples. It is important to carefully analyze and distinguish between host and microbial sequences to avoid misinterpretation of results.

## 8. Conclusion

Emerging evidence have highlighted the possibility of a brain microbiome and its role in the development of neurodegenerative diseases, mainly AD and PD, primarily through neuroinflammation and neurotoxicity. Bacteria, such as *C. pneumoniae*, *B. burgorferi*, *H. pylori*, and *C. acnes*, appear to play a role in the development of neurodegenerative diseases. Fungi from the genera *Candida* and *Malassezia* have also been found in the brains of neurodegenerative disease patients. However, it is important to note that several studies have reported negative results in the brains of AD, PD, and, even, normal patients.

Multiple theories have risen attempting to explain the methods utilized by microorganisms to invade the central nervous system. Prominent theories include inflammation-induced BBB disruption, bacterial invasion of the BMECs, and GALT-mediated bacterial translocation. However, evidence supporting these hypotheses are limited, and further studies are needed to verify them. Several limitations also face the brain microbiome theory. Some have postulated that exogenous DNA contamination and off-target amplification of host DNA may explain the contradictory data seen in the literature. Unfortunately, human studies assessing the effectiveness of antimicrobial treatment in neurodegenerative diseases have failed to demonstrate any impact on disease progression. Overall, the brain microbiome theory is emerging as a prominent theory in the pathogenesis of neurodegenerative disease development. However, it is crucial to interpret these results with caution until further studies are reported.

## Author contributions

TA, AA, and BS drafted the manuscript. AO critically revised the manuscript and conceptualized the topic of the manuscript. All authors contributed to the article and approved the submitted version.
